# Impact of early-life human microbiota on the murine host metabolome: insights from a two-generation HMA mouse model and implications for allergic disease

**DOI:** 10.1186/s12866-025-04321-9

**Published:** 2025-09-16

**Authors:** Ymke A. de Jong, Rana M. Seren, Vida Ramšak Marčeta, Antonio Checa, Dagbjort H. Petursdottír, Isabella Badolati, Claudia Moeckel, Omneya Ahmed Osman, Eva Hell, Douglas L. Huseby, Diarmaid Hughes, Craig E. Wheelock, Sarahi L. Garcia, Klas I. Udekwu, Khaleda R. Qazi, Eva Sverremark-Ekström

**Affiliations:** 1 Department of Molecular Biosciences, The Wenner–Gren Institute, Stockholm University, Stockholm, Sweden; 2 Unit of Integrative Metabolomics, Institute of Environmental Medicine, Karolinska Institute, Stockholm, Sweden; 3 Department of Materials and Environmental Chemistry, Stockholm University, Stockholm, Sweden; 4 Department of Medical Biochemistry and Microbiology, Uppsala University, Uppsala, Sweden; 5 Department of Respiratory Medicine and Allergy, Karolinska University Hospital, Stockholm, Sweden; 6 Department of Ecology, Environment and Plant Sciences, Science for Life Laboratory, Stockholm University, Stockholm, Sweden; 7 Institute for Chemistry and Biology of the Marine Environment, School of Mathematics and Science, Carl Von Ossietzky Universität Oldenburg, Oldenburg, Germany; 8 Department of Biological Sciences, Bioinformatics and Computational Biology Program, University of Idaho, Moscow, ID USA

**Keywords:** Allergy, Infant, Microbiota, Metabolome, Immune profile, Liver, Intestinal tissue, Human microbiota-associated mouse model

## Abstract

**Introduction:**

Human microbiota-associated (HMA) models are used to allow in vivo studies of the human gut microbiome and its effects on host physiology. In particular, alterations in early life microbiota have been linked to allergy development during childhood. In this study, we investigated how pools of human microbiota collected from infants with different allergy risk, thrive in mice and their offspring, as well as how they influence the host metabolome.

**Method:**

We used a two-generation HMA mouse model in which dams were colonized with human feces from three groups of infants (*n* = 19, samples collected during the first 8 weeks of life). In two of the groups, all infants had a strong hereditary risk for allergic disease (*n* = 12), but only 6 of them developed allergy before 2 years of age. In the third group, which was used as a control, none of the infants had allergic heredity or developed allergy (*n* = 7). Microbiota trajectories were followed from inoculation to mouse offspring, and metabolic profiles were monitored in several intestinal organs as well as in the serum of the murine offspring.

**Results:**

The human microbiota adapted to the murine host but still presented distinct compositional features, reflecting the original inoculated samples. These microbial differences were mirrored in the mouse offspring metabolome, with group-associated patterns in sphingolipids, acylcarnitines and tryptophan metabolites. Furthermore, the metabolic profiles of the mouse offspring aligned with those observed in fecal water preparations from the corresponding human infant fecal samples.

**Conclusion:**

Our findings highlight the significant impact of early-life microbiota on the host metabolome and show that our two-generation HMA model is suitable for studying microbiota‒metabolome relationships relevant to humans. The differences in microbiota‒metabolome correlations between individuals who develop or do not develop allergic disease suggest that an allergic predisposition might be more multifaceted than previously believed.

**Supplementary Information:**

The online version contains supplementary material available at 10.1186/s12866-025-04321-9.

## Background

Host physiology and the gut microbiome influence each other, and numerous studies have linked specific microbial compositions or specific bacteria to a variety of diseases, including allergies [1–7]. Despite this, establishing a causative link between a dysbiotic gut and disease in humans is often difficult. Different types of murine models, ranging from germ-free to wildling systems [8], have been instrumental in characterizing the interactions between the gut microbiota and disease. To study a microbiota relevant to humans, human microbiota-associated (HMA) mouse models have been established, in which germ-free (GF) mice are inoculated with fecal matter obtained from humans. The validity of one-generation HMA models is discussed, as the symbiotic relationship between the host and its microbiota is shaped by coevolution, creating a host-specific relationship, which can lead to inconsistent engraftment of the human microbiota into GF mice [9–13]. Additionally, the lack of an early microbiota is believed to be the main reason behind the compromised immune system in GF mice, as these two entities normally develop together. Colonizing GF mice with human microbiota after weaning, could lead to a compromised maturation of the immune system and other physiological functions [12, 14–16].

The early life gut microbiota is of particular interest for human health, as it influences immune development and maintains tolerogenic immune functions [17, 18]. Early-life microbiota characteristics differ in children who subsequently develop allergy during childhood, as reported in many studies [19–22]. A delayed and/or impaired gut microbiota maturation in early life, as well as reduced abundance of different species of e.g., *Akkermansia*, *Bifidobacterium*, *Faecalibacterium*, and *Lactobacillus*, are features associated with an increased allergy risk [23]. Still, results differ between studies and regions, illustrating the complexity of the gut microbiota and its role in immune regulation and disease development. It is more likely that a network of microbes and their functional features, rather than single specific taxa, drive immunoregulatory or inflammatory signatures that either protect or promote allergic immune profiles [7].

Despite extensive research, there are still gaps in knowledge regarding which mediators are involved in the gut microbiota-host physiology/immune cross-talk, especially in various tissues and/or organs. Many different microbial- and host-derived factors have been suggested to play a role and have been verified in model systems, including *Bacteroides fragilis* polysaccharide A [24], short-chain fatty acids (SCFAs), bile acids, vitamins and aryl hydrocarbon receptor (AhR) ligands [25, 26]. These metabolites are also implicated as mediators of disease development and/or protection, for example, in allergies [1, 27–29]. As the gut microbiota contributes significantly to the host metabolome, it can be described as a signature of host interactions with the environment. Notably, because both metabolites and metabolic pathways are conserved between species, it is possible to extrapolate metabolome-derived findings from mice to humans [30, 31].

Here, we introduce a novel two generational HMA model that is suitable for studying early life microbiota‒metabolome relationships relevant to humans. To avoid the pitfalls with a one-generation HMA model, we colonized adult GF mice with pooled fecal matter, collected during the first weeks of life from three groups of human infants [32, 33]. We then investigated microbiota characteristics of the human infant fecal inoculates, dam cecal content and feces, as well as offspring cecal- and large intestinal content, by 16S rRNA amplicon sequencing to track the engraftment and vertical transmission from dam to offspring. Furthermore, we explored the metabolic profile in the serum, intestinal organs (liver, intestinal tissue, and abdominal fat) and intestinal content from the mouse offspring, as well as in the human infant fecal inoculates, to identify unique and overlapping signatures of the tissues as well as microbiota dependence. We also show that the microbiota‒metabolome axis is different in individuals who develop or do not develop allergic disease, suggesting that an allergic predisposition might be more multifaceted than previously believed.

## Methods

### Mice

Germ-free (GF) C57BL/6 mice were kept in isolators and fed sterile R36, Lactamine Chow (Lantmannen, Sweden) at the Core Facility for Germfree Research at the Karolinska Institutet, Stockholm, Sweden. To ensure sterility and GF status, fecal samples from all GF isolators were tested weekly; cultures on blood agar plates (aerobic and anaerobic growth), CLED plates (cysteine-, lactose- and electrolyte-deficient) and Sabouraud dextrose agar (Oxoid) plates were monitored weekly.

### Human infant fecal samples

Infant fecal samples were obtained from a well-described prospective cohort to study how allergic heredity and environmental factors influence allergy development from early life until adulthood [33, 34]. The fecal samples used for the current study were collected during weeks 2–8 after birth from healthy, full-term, fully breastfed infants (*n* = 19) and stored at −80 °C until further use. The fecal samples were pooled as follows: 6 children with two allergic parents (high hereditary allergy risk), who did not develop allergy at the age of 2 years, are referred to here as non-allergy-associated microbiota (non-AAM); 6 fecal samples from children with high hereditary allergy risk, who did develop allergy at the age of 2 years, are referred to here as allergy-associated microbiota (AAM); and 7 children who had two non-allergic parents (low hereditary allergy risk), who did not develop allergy at the age of 2 years, are referred to as the control group (control).

### Experimental setup and sample collection

**Fig. 1:**
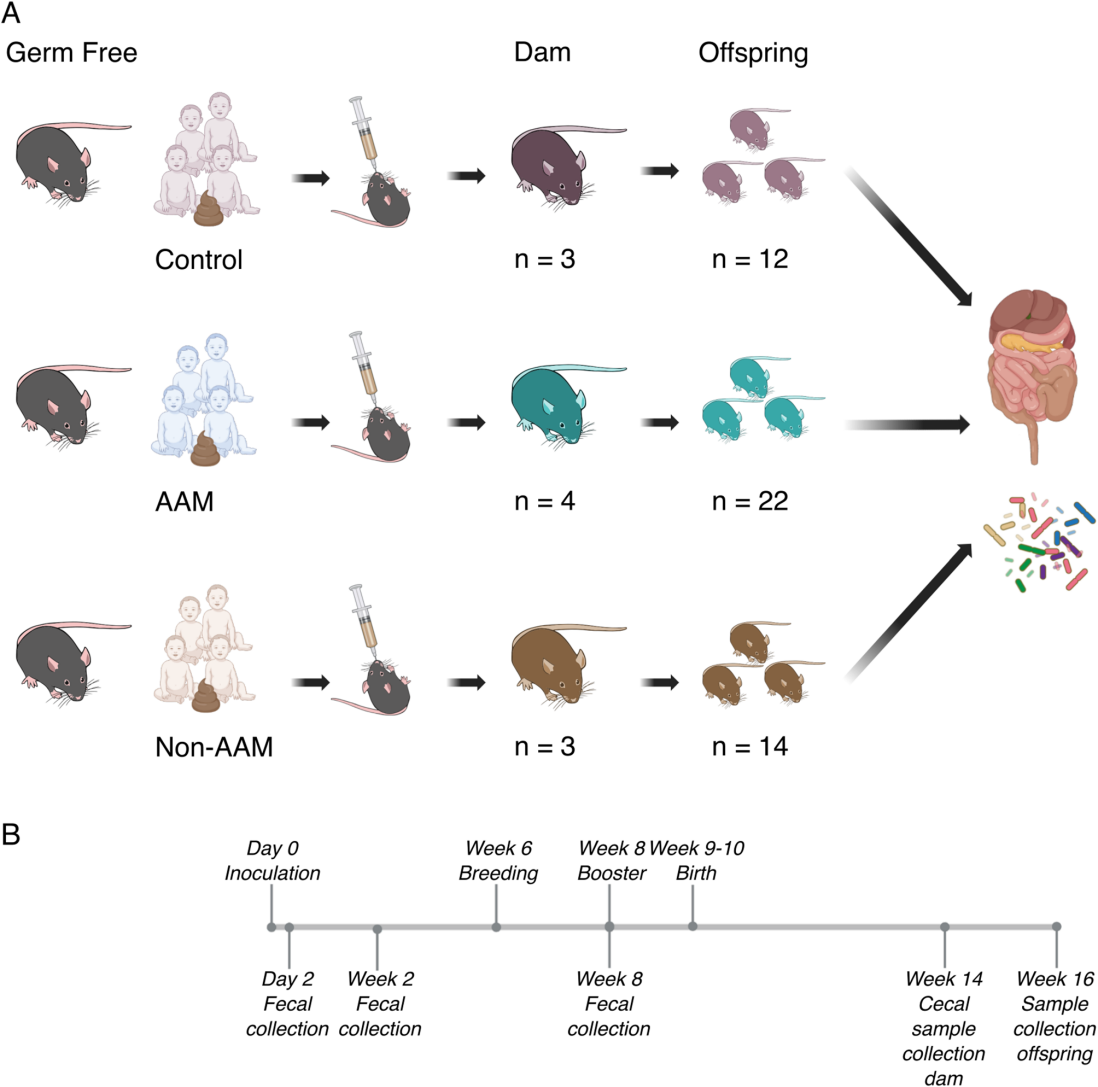
Human microbiota-associated mouse model and timeline of experimental processing and sample analysis. **A** Germ-free mice were inoculated with 3 pools of human infant microbiota from three groups of infants (control, n = 7), (AAM, n = 6) or (non-AAM, n = 6). Dams (3, 4, and 3 respectively) were allowed to breed, and after a booster inoculation, offspring was born (control n = 12, AAM n = 22, non-AAM n = 14). Dam fecal (control n = 7, AAM n = 14, non-AAM n = 7) and cecal (control n = 3, AAM n = 4, non-AAM n = 3) samples were analyzed for microbiota characterization of the dams, while offspring cecal (control n = 12, AAM n = 22, non-AAM n = 14) and large intestine (control n = 11, AAM n = 22, non-AAM n = 13) samples represented the offspring microbiota. Intestinal content, liver, intestinal tissue, serum and adipose tissue were collected from the offspring and a metabolic screening was performed by LC–MS/MS (n = 6 per group and compartment). **B** Timeline of mouse experiment

To generate the human microbiota-associated (HMA) mouse model, the GF mice were colonized with non-AAM (dams, *n* = 3), AAM (dams, *n* = 4), or control (dams, *n* = 3) microbiota pools by gavage, as depicted in Fig. 1A (previously described [32]). Briefly, fecal samples were homogenized in sterile phosphate-buffered saline (PBS) (0.3 g/ml) and 0.1 ml of fecal PBS solution was fed orally to the mice at the age of 6 weeks. After 6 weeks, the mice were bred. A booster was given to the mouse dams on gestational days 15–17. All groups of offspring had between 54–63% males. Each GF line was bred and maintained in at least two different isolators. Fecal samples from the dams and intestinal content from the dams and offspring were collected as described below and are shown in Fig. 1B. Fecal samples from the dam HMA mice were collected at 2 and 14 days post gavage and 2 days post booster, prior to giving birth. Cecal samples were collected from the dams 14 weeks after colonization. For the offspring, content from cecum and large intestine samples were collected at euthanasia at 7–8 weeks of age (week 16 of the study). The mice were euthanized by cervical dislocation without anesthesia, a method approved by the North Stockholm Ethics Committee (permission N31/14). Liver and intestinal tissues, abdominal fat and plasma were also collected from the offspring at euthanasia and kept frozen at −80 °C until further analysis. In parallel, GF samples were collected from 3–20-week-old male and female mice at the same facility.

###  16 s rRNA amplicon analysis

Microbial DNA was extracted using ZR Fecal DNA MiniPrep (Zymo Research) and purified with AMPure XP Beads (Beckman Coulter) according to the manufacturer’s instructions. Amplicon sequencing of 16S rRNA genes from the HMA and infant samples was performed as previously described [32]. Briefly, the v3—v4 region of the 16S rRNA gene was amplified using barcoded 341 F and 805R primers. The PCR products were pooled at equimolar concentrations, followed by adapter ligation using TruSeq DNA PCR-free LT Library Preparation Kit (Illumina, San Diego, CA, USA). Paired-end sequencing (2 × 300 bp) was performed on the Illumina MiSeq platform using the MiSeq V3 reagent kit spiked with 10% PhiX (Illumina, San Diego, CA, USA). For the GF mice, the 16S rRNA gene was amplified by using 16S metagenomic sequencing library preparation protocol from Illumina. Briefly, the 16S rRNA gene was first amplified with non-barcoded 341 F and 805R primers and then purified by AMPure XP Beads (Beckman Coulter). This was followed by a second PCR amplification with Nextera XT index primers (Nextera XT index kit FC-131–1001 or FC-131–1002). The barcoded PCR amplicons were purified with AMPure XP Beads, and quality checked on an Agilent 2100 BioAnalyzer (Agilent Technologies, Palo Alto, CA, USA) prior to pooling. Amplicons were denatured, spiked with 5% PhiX and sequenced on the Illumina MiSeq platform according to the manufacturer’s protocol using the MiSeq V3 reagent kit.

### Fecal water preparation

400 mg of infant feces were diluted in sterile PBS (25% w/v) and vigorously mixed in a FastPrep-24 instrument (MP Biomedicals European HQ) for 1 min at 6.5 m/s. This was followed by 20 min of centrifugation at 1 600 g at 4 °C. The supernatant was centrifuged again for 10 min at 14 000 g and 4 °C. The obtained supernatant was then diluted in an equal volume of RPMI and subsequently sterile filtered through a 0.45 µm filter followed by a 0.22 µm filter and stored at −80 °C until use.

### In vitro stimulation of peripheral blood mononuclear cells (PBMCs) with fecal water

Cryopreserved PBMCs from healthy donors were thawed and washed three times with warm RPMI-1640. The cells were counted with an automated cell counter (Countess 3, Life Technologies Europe BV), and viability was determined with Trypan Blue staining. The cells were subsequently resuspended at a concentration of 2 × 10^6^ cells/mL in cell culture medium, which consisted of RPMI-1640 supplemented with 20 mM HEPES, 100 U/mL penicillin, 100 µg/mL streptomycin, 2 mM L-glutamate (all from GE Healthcare Life Sciences, Cytiva, Chicago, IL, USA), and 10% heat-inactivated fetal calf serum (Sigma Aldrich, Merck, Darmstadt, Germany). PBMCs were seeded in a flat-bottom 24-well tissue culture plate, and left unstimulated or stimulated with 1% or 10% fecal water from the AAM or non-AAM infant feces. The cells were then incubated for 48 h at 37 °C and 5% CO_2_ (Costar, Cambridge, UK). After 48 h, the supernatants were collected and stored at −80 °C until analysis for cytokines by ELISA.

### Detection of cytokines by sandwich ELISA

Sandwich ELISA was performed for the detection of IL-6, IL-10, IL-13, IL-17A, IL-22 and IFNγ (MABTECH, Stockholm, Sweden) in the culture supernatants according to the manufacturer’s instructions.

### Sample preparation for metabolomics and sphingolipid analysis

Tissue samples and intestinal content were placed on dry ice, followed by the addition of 40 μL of ice-cold methanol per mg of tissue. 50 mg of 1 mm ZrO beads and 2 × 2 mm ZrO beads (Techtum, Nacka, Sweden) were added to each sample, and homogenized on a Fisherbrand Bead Mill 24 (1 × 15 s cycle, strength = 6). For the intestinal tissue, homogenization was performed twice. The samples were then placed in an ultrasonic bath and sonicated for 15 min on ice. Next, the samples were centrifuged at 10 000 *g* for 15 min and finally, 90 µL of the supernatant and 10 μL of the sphingolipid internal standard mixture were combined in an LC‒MS vial equipped with a 300 μL insert.

For the serum samples, 10 µL of sphingolipid internal standard mixture was added to 10 µL of sample, followed by 100 μL of LC‒MS methanol. The samples were vortexed for 5 s, placed in an ultrasonic bath and sonicated for 15 min on ice. Next, the samples were centrifuged at 10 000 *g* for 15 min and 80 μL of the extract was transferred to an LC‒MS vial equipped with a 300 μL insert.

### LC‒MS/MS analyses

*Sphingolipid analyses* Separation of sphingolipids was performed on an ACQUITY UPLC System using a sample manager cooled to 8 °C (Waters Corporation, Milford, MA, USA). Mobile phases A and B consisted of 5 mM ammonium formate/0.2% formic acid in water (A) and methanol (B), respectively. For separation, an ACQUITY Premier CSH C18 FIT Column (130 Å, 1.7 µm, 2.1 mm X 100 mm) with a Vanguard FIT cartridge (Product Number: 186009464; Waters Corporation) was used. The UPLC method had a flow rate of 350 μL/min and a column temperature of 45 °C. The chromatographic gradient was as follows: 0.0 min → 1.0 min, 80% B (isocratic range); 1.0 min → 2.5 min, 80% → 85% B (linear increase); 2.5 min → 3.0 min, 85% → 97% B (linear increase); 3.0 min → 9.0 min, 97% → 100% B (linear increase); 9.0 min → 13.0 min, 100% B (isocratic range); and 13.0 min → 13.1 min, 100% → 80% B (linear decrease). The column was then conditioned for 0.5 min until the next injection. A sample volume of 2.5 µL was injected for each sample. The acquisition was performed on a Waters Xevo® TQ-S system equipped with an Electrospray Ion Source (ESI) and ScanWave™ collision cell technology operating in positive mode. A class‒specific selected reaction monitoring (SRM) transition for each sphingolipid was used. The analysis was performed in positive mode, with a capillary voltage of 3 kV. The desolvation gas flow rate and temperature were set at 650 L/h, and 550 °C, respectively.

*Polar metabolites* were analyzed using the same UPLC‒MS/MS system used for the sphingolipids applying a previously published method with minor modifications [35]. Briefly, mobile phases A and B consisted of 20 mM ammonium formate/0.1% formic acid in water (A) and 0.1% formic acid in acetonitrile (B), respectively. For the separation, an ACQUITY Premier BEH amide column (130 Å, 1.7 µm, 2.1 mm X 100 mm) with a Vanguard FIT cartridge (Product Number: 186009457, Waters Corporation) was used. The UPLC method had a flow rate of 400 μL/min, and the column temperature was held at 30 °C. The chromatographic gradient was as follows: 0.0 min → 1.5 min, 95% B (isocratic range); 1.5 min → 14.0 min, 95% → 55% B (linear decrease); 14.0 min → 14.2 min, 55% → 50% B (linear decrease); 14.2 min → 16.5 min, 45% B (isocratic range); 16.5 min → 17.0 min, 45% → 95% B (linear increase). The column was then conditioned for 5.5 min until the next injection. A sample volume of 2.5 µL was injected for each sample. The acquisition was performed on a Waters Xevo® TQ-S system equipped with an ESI and ScanWave™ collision cell technology operating in the positive mode. Optimized SRM transitions were acquired for each compound. The analysis was performed in positive mode, with a capillary voltage of 3 kV. The desolvation gas flow rate and temperature were set at 1000 L/h, and 550 °C, respectively.

### SCFA sample preparation

Samples and standards were prepared as previously described [36] as follows: 100 mg of liver or cecum tissue was homogenized with a cell strainer, and 10% isobutanol/water was added. The samples were further homogenized by vortexing and subsequently centrifuged at 17 000 g for 7 min. 675 μL sample supernatant, 21.7 μL of the internal standard, 3,2-methylhexanoic acid (Sigma Aldrich), 125 μL 20 mM NaOH and 400 μL chloroform (Honeywell, Riedel–de Haen, Germany) were mixed and centrifuged at 17 000 g for 4 min. A total of 400 μL of the aqueous phase was transferred to a tube with one 1‒2 mm boiling chip (Supelco, Merck). Subsequently, 80 μL of 10% isobutanol/water and 100 μL of pyridine were added and the volume was adjusted to 650 μL with MQ H_2_O. The samples were stored at −80 °C or derivatized immediately.

A dilution series of standards was prepared using a volatile‒free acid mixture (CRM46975, Sigma Aldrich). Each standard tube contained 125 μL 20 mM NaOH, 80 μL 10% isobutanol (Merck) and 100 μL pyridine (Merck), volatile free acid mixture, a boiling chip and Milli‒Q H_2_O up to a volume of 650 µL. A total of 21.7 µL of internal standard was added before derivatization.

The standards and samples were derivatized by adding 50 μL of isobutyl chloroformate (Sigma Aldrich) followed by 150 μL hexane. After vortexing and centrifugation, the aqueous phase was transferred into a glass vial with a crimp top. The samples were stored at −20 °C or immediately analyzed.

### SCFA quantification

GC‒MS measurements were performed on a Varian 450 GC system coupled to a triple quadrupole mass spectrometer Varian 320-MS. A VF‒5 column (30 m × 0.25 mm, 0.5 μm; Agilent Technologies, Palo Alto, CA, USA) was used for separation of the analytes. Supplementary Tables 1 and 2 show the settings used for the GC‒MS analysis. Peak area-based quantification against a set of 6 calibration standards was performed with Bruker MS Workstation version 7.

### Bioinformatics and statistical analysis

HMA mice and infant sequence results were demultiplexed using UltraPlex (Python, v 1.2.6) [37]. Quality, trimming and maximum expected error parameters were determined on the basis of results from fastqc (Java, v 0.12.0) [38], multiqc (Python, v 1.14) [39] and figaro (Python, v 1.1.2) [40]. Amplicon sequence variants (ASVs) were determined using DADA2 (R package, v1.26) [41]. Non default settings are shown in Supplementary Table 3. Taxonomy was assigned using SILVA database (v138.1) [42]. For the GF samples, the sequences assigned as Proteobacteria mitochondria were compared against the NCBI non-redundant nucleotide database via BLASTn (BLAST, ncbi). Further microbiota data analysis was performed in R (v4.3.3), using *phyloseq* (v1.44.0) [43], *rstatix* (v0.7.2)*, vegan* (v2.6.4) [44], *ggpubr* (v0.6.0)*, Deseq2* (v1.44.0) [45], or *PCAtools* (v2.12.0) [46]. For calculations of α-diversity (richness) and relative abundance plots, the samples were rarefied to 5 570 reads, and samples with < 5 570 read were removed (Supplementary Fig. 1). Relative abundance counts were used to plot the β‒diversity, and differential abundance was calculated from the raw counts. Fecal and cecal samples from the dams were analyzed together and labeled “dam”, whereas large intestine and cecal samples from offspring were pooled as “offspring”.

Metabolomic data were analyzed using *PCAtools* (v2.12.0) and *vegan* (v2.6.4) for principal component analysis and statistics. Missing values were replaced by 0.25 × minimum obtained value. Owing to the design of the study, differential abundance was tested by t‒test (rstatix v0.7.2), and as we looked at the overview of the metabolomic pattern, no false discovery rate (FDR) adjustment were performed. Visualization of all the data was performed using *VennDiagram* (v1.7.3)*, gplots* (v3.1.3.1) and *ggplot2* (v3.4.4).

## Results

### Fecal water from non-AAM and AAM inoculates have divergent metabolomes and induce distinct immune profiles in human peripheral immune cells

**Fig. 2:**
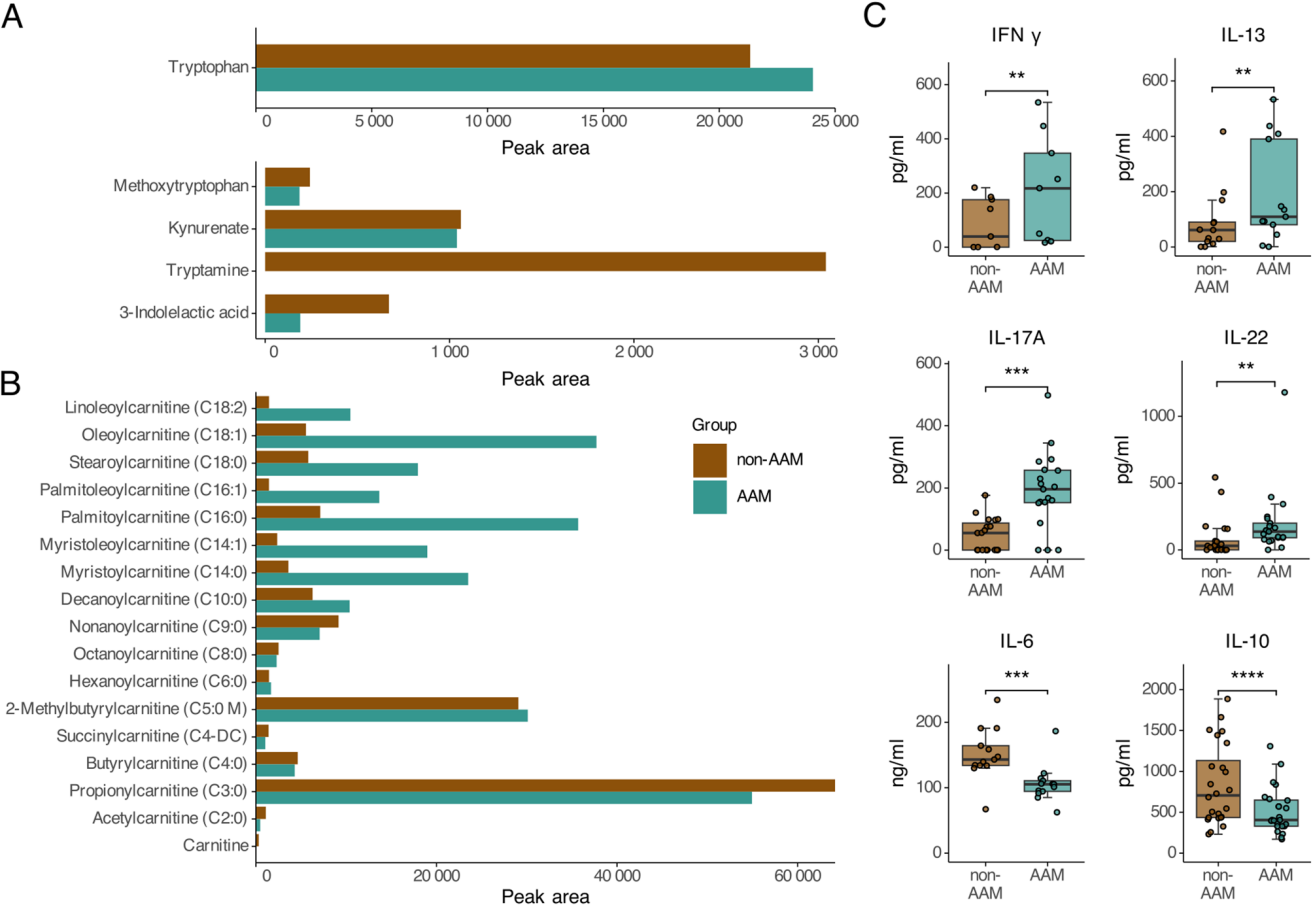
Human infant fecal waters differ with respect to tryptophan and acylcarnitine profiles and induce different cytokine profiles in PBMCs. **A** Tryptophan and several of its metabolites in human infant fecal water analyzed by UPLC-MS/MS. Average of 2 technical replicates in each group. **B** Acylcarnitines in human infant fecal water analyzed by UPLC-MS/MS. Average of 2 technical replicates in each group. **C **Cytokines secreted by PBMCs, stimulated with 10% (IFNγ, IL-13, IL-17A and IL-22) or 1% (IL-6 and IL-10) human infant fecal water. (Paired Wilcoxon signed rank test; ** *p* ≤.01, *** *p* ≤.001, and **** *p* ≤.0001, *n* = 9—19)

To confirm a relationship between the microbiome, metabolome and an immune profile relevant for allergy development, we prepared fecal water samples from two of the three fecal pools available from human infants (non-AAM and AAM), as described in the Materials and Methods. These fecal water preparations were analyzed for a large group of polar metabolites, as several of these compounds have been connected to immune function and tolerance via the aryl hydrocarbon receptor (AhR). Indeed, we observed that several polar metabolites differed between the non-AAM and AAM fecal water samples (Supplementary Fig. 2). Although tryptophan itself was slightly lower in fecal water from the non-AAM compared to the AAM group, the levels of some tryptophan metabolites, including tryptamine and 3-indolelactic acid (ILA), were higher (Fig. 2A). In contrast, long (> C10) acylcarnitines were more abundant in the AAM fecal water (Fig. 2B).

Next, we investigated whether these metabolome differences impact immune profiles. Accordingly, we stimulated human PBMCs with fecal water from non-AAM or AAM inoculates. Marked activation of type-1 (IFNy), type-2 (IL-13), and type-3 (IL-17, IL-22) responses was observed in the cells stimulated with fecal water from the AAM inoculum. In contrast, there was a bias toward an innate response with regulatory features (*e.g.,* IL-10 and IL-6) from the cells stimulated with fecal water from the non-AAM inoculum (Fig. 2C).

### Initial composition of the human microbiota defines microbiota trajectories in an HMA model

Based on the above, we used our HMA model, to study the relationship between the gut microbiota and metabolome in vivo. To evaluate the validity of the model, *i.e.*, how the human infant microbiota thrives in mice, we followed microbiota trajectories from the inoculum to the mouse dams and murine offspring. We inoculated germ-free mice with the three microbiota pools described in the Methods and illustrated in Fig. 1. Fecal pools from 6‒7 individuals per group were used to reduce the interpersonal variation normally observed in humans. Mean relative abundance counts were used to visualize differences in the mouse groups over time.

**Fig. 3:**
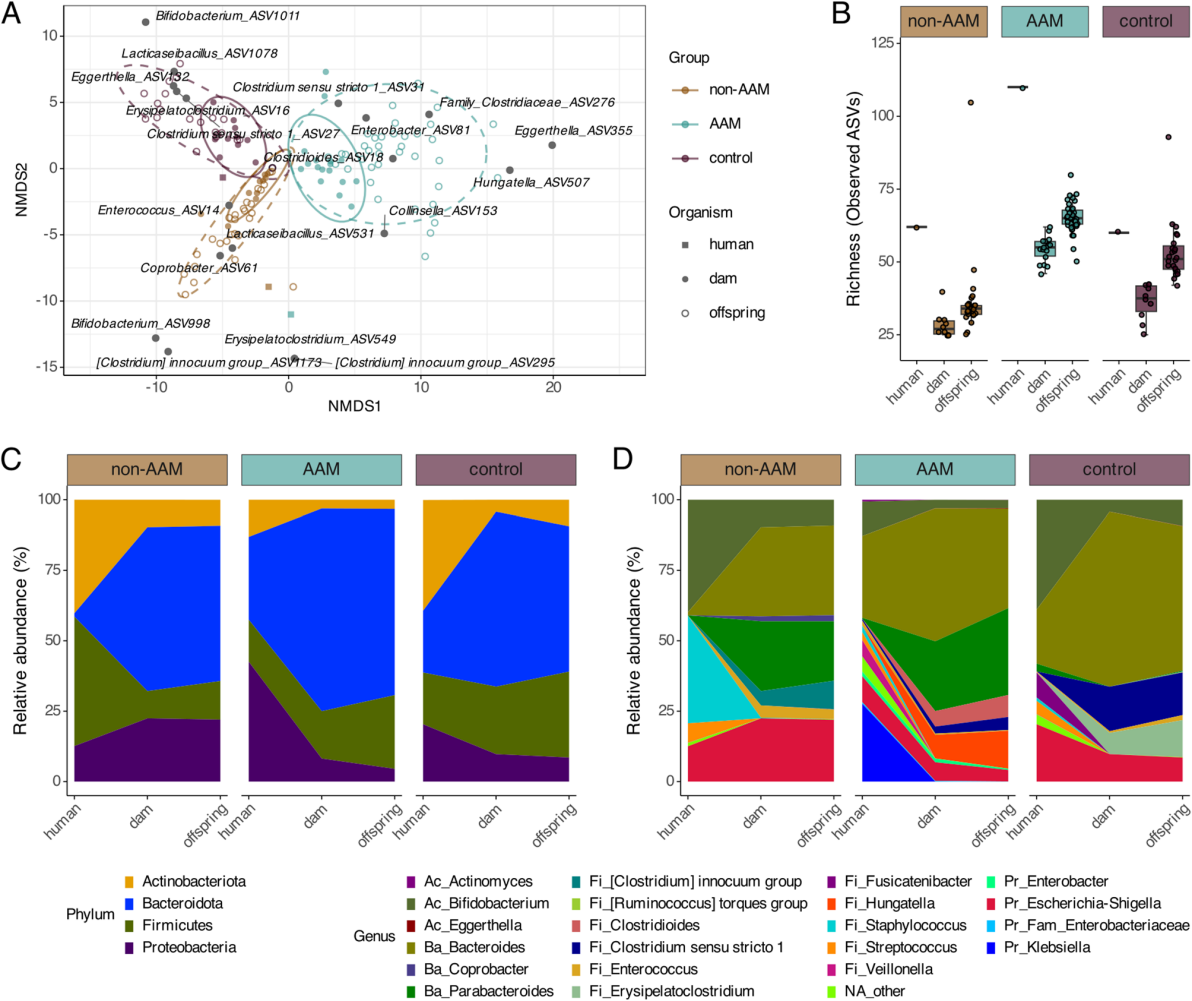
Intestinal microbiota trajectories are influenced by human to mouse transition in a group-dependent way. **A** Non-metric multidimensional scale (NMDS) plot showing β-diversity based on normalized microbiota data and robust aitchison dissimilarity (stress value = 0.1216558). PERMANOVA testing (Adonis2, 999 permutations) showed that all three groups significantly differ from each other (*R*^*2*^ =.33, *p* =.001), both in the dam (*R*^*2*^ =.2905, *p* =.001) and in the offspring (*R*^*2*^ =.20817, *p* <.001). There was a significant difference between the dam and offspring cluster for non-AAM and AAM respectively (*R*^*2*^ =.01812, *p* =.003, pairwise.adonis2), as well as their variance within the clusters (*p* < 0.001, anova). Bacterial genera shown based on highest abundant ASVs of those genera. **B** α-diversity (richness), displayed as observed ASVs, on rarefied data divided by group, from human infant inoculate to dam followed by offspring. **C**, **D** Mean relative abundance of gut microbes at phylum (**C**) and genus (**D**) level for human, dam and offspring samples. Most abundant taxa, representing a total 99% of taxa are included, remaining taxa are labelled as “NA_other”

We observed that the microbiota in the non-AAM and AAM groups largely shifted after introduction into the mice, while the control group inoculum did not clearly separate from the murine samples (Fig. 3A). Even though the microbiota adapted to the new host environment, each group followed its own unique trajectory in the mice (PERMANOVA, *R*^*2*^ = 0.33, *p* = 0.001), demonstrating that the murine microbiota depends on the initial composition of the human infant inoculum. The offspring microbiota continued to adjust to the new host environment; however, all three groups of offspring were more similar to their respective dams than to each other. The AAM samples clustered in the direction defined by *Hungatella, Enterobacter, Clostridioides, Collinsella* and *Clostridiaceae*, whereas the control and non-AAM samples were more closely related to *Bifidobacterium, Lacticaseibacillus, Enterococcus,* and *Clostridium innocuum group* (Fig. 3A).

Richness showed the same pattern in all three groups, with an initial decline of ~ 50% after introduction into the mice (Fig. 3B). During the transition from the dam to the offspring, previously undetected ASVs bloomed and recovered to above the detection threshold, thereby increasing the estimated richness. In total, 45%, 52% and 57% of the taxa in the inoculum were recovered from the murine non-AAM, AAM and control samples, respectively. These taxa represented between 51 and 68% relative abundance in each murine sample; the remaining taxa were most likely below the detection limit in the inoculates.

From a compositional perspective, the non-AAM inoculum was dominated by Actinobacteriota and Firmicutes, while the AAM inoculum consisted mainly of Proteobacteria and Bacteroidota but had a low proportion of Actinobacteriota. The control inoculum had a relatively even distribution among the four main phyla. After establishment in the mice, the microbiota in all three groups became more similar at the phylum level, all with a dominance of Bacteroidota (Fig. 3C, Supplementary Fig. 3A). In general, the results for the offspring samples were very similar to those for the dam samples, with an overall increase in Firmicutes in the offspring, which was compensated by a decrease in Bacteroidota and Proteobacteria.

The differences observed at the phylum level could be explained by some of the most abundant genera (Fig. 3D, Supplementary Fig. 3B). Actinobacteria were represented by only one genus, *Bifidobacterium,* while Firmicutes exhibited high diversity in all three groups. The Bacteroidota were represented mainly by *Bacteroides* in the control group, whereas the non-AAM and AAM groups presented an increasing abundance of *Parabacteroides* in the mice. The Proteobacteria in both the non-AAM and control groups were mainly represented by *Escherichia-Shigella*, while the AAM inoculum was dominated by *Klebsiella*, which diminished in the mice and was replaced by *Escherichia-Shigella*. All three groups had *Streptococcus* and *Enterobacter* in the inoculum, but these genera did not establish themselves in the mouse model. *Staphylococcus*, which was highly abundant in the non-AAM inoculum, also decreased radically in all three groups of mice.

Upon sequencing the GF mouse fecal samples for viable but nonculturable bacteria, we detected 16S rRNA gene sequences; however, at least 97% of each sample was mouse mitochondrial DNA, and none of the ASVs in the GF mice were detected in the humanized mice (Supplementary Fig. 4).

### Sphingolipid profiles are correlated with the gut microbiota composition

Because sphingolipid profiles reflect environmental signals and are strongly associated with immune function and allergic diseases, we evaluated the murine metabolome by investigating the sphingolipid composition. Sphingolipids were analyzed in several abdominal compartments of the offspring (liver, intestinal tissue, adipose tissue, intestinal content and serum) and in the infant inoculates (Supplementary Fig. 5).

**Fig. 4:**
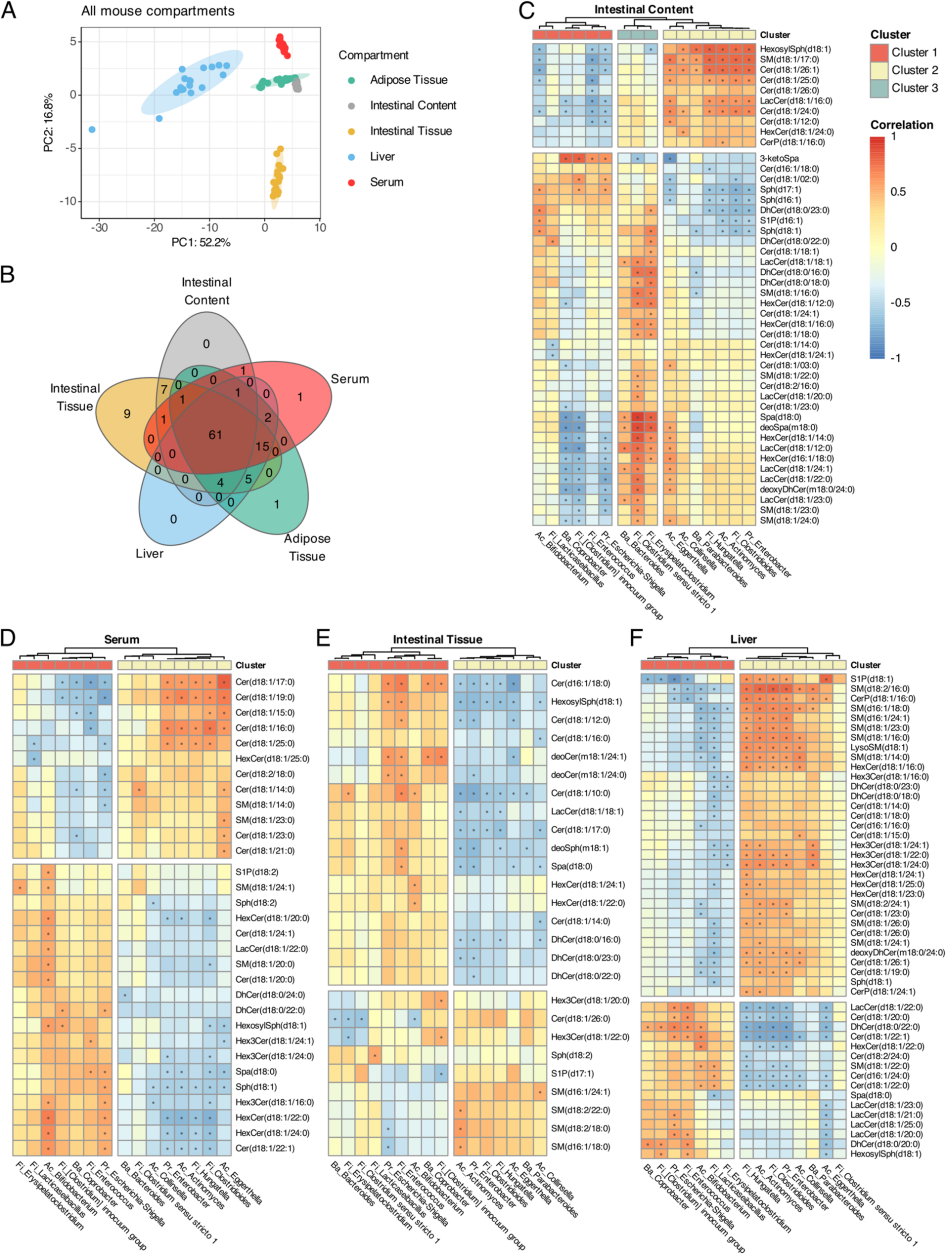
Sphingolipid composition in murine intestinal-, liver-, and serum compartments are microbiota dependent. **A** Principal component analysis (PCA) based on the sphingolipid results on centred and scaled data, for all mice offspring compartments (*n* = 18). **B** Venn diagram showing number of detected sphingolipids found in each compartment. **C**-**F** Spearman correlation matrices of bacteria from the mouse offspring at genus level with sphingolipids in each measured mouse compartment. Only metabolites and bacteria with significant (*p* ≤ 0.05) correlations are shown. Optimal number of clusters are determined using Silhouette method

The sphingolipid profile in the intestinal content clustered closely with that in the serum and adipose tissue, while the profiles in the liver and intestinal tissue separated from the other compartments (Fig. 4A). An initial screening revealed 120 reported sphingolipids. A total of 61 metabolites were detected in all the murine compartments, including the intestinal content, and 15 were detected only in the murine organs, excluding the intestinal content (Supplementary Fig. 5). The intestinal content had no unique metabolites, whereas the intestinal tissue had nine unique hits (Fig. 4B).

Next, we tested for correlations between the microbiota and sphingolipids, which revealed several significant associations. In all compartments, different bacterial genera clustered together. *Escherichia-Shigella*, *Clostridium innocuum* group, *Coprobacter, Enterococcus, Lacticaseibacillus,* and *Bifidobacterium* formed cluster 1. *Eggerthella*, *Hungatella, Actinomyces, Enterobacter, Collinsella, Parabacteroides,* and *Clostridioides* formed cluster 2. The intestinal content contained a third cluster of *Erysipelactoclostridium, Clostridium *Sensu stricto* 1,* and *Bacteroides*, which was divided between clusters 1 and 2 in the other compartments (Fig. 4C-F).

In the intestinal content, cluster 1 was negatively correlated with a group of d18:1 ceramides, whereas cluster 2 bacteria were positively correlated with these sphingolipids. The third cluster was positively correlated with lactosylceramides (LacCers), hexosylceramides (HexCers), and dihydroceramides (DhCers). 3-Ketosphinganine (3-ketoSpa), a precursor for sphingolipids, had a strong positive correlation with *Coprobacter* and the *Clostridum innocuum* group and a strong negative correlation with *Eggerthella*. Sphingosine-1-phosphate (S1P(d16:1)), an important bioactive lipid involved in signaling, was negatively correlated with several bacteria in cluster 2 (Fig. 4C). In the serum, a group of short and long d18:1 ceramides (Cer(d18:1)) correlated positively with cluster 1 bacteria and negatively with several cluster 2 bacteria (Fig. 4d). In the intestinal tissue, bacterial cluster 2 correlated mainly negatively with a range of ceramides (Fig. 4E). In the liver, we observed strong positive correlations between the four main bacteria in cluster 2 and both short and long sphingomyelins (SM), S1P(d18:1), long trihexosylcermides (Hex3Cers), and long ceramides (Fig. 4F).

### Major differences in the sphingolipid profiles between non-AAM and AAM mice in intestinal content as well as in the liver

**Fig. 5:**
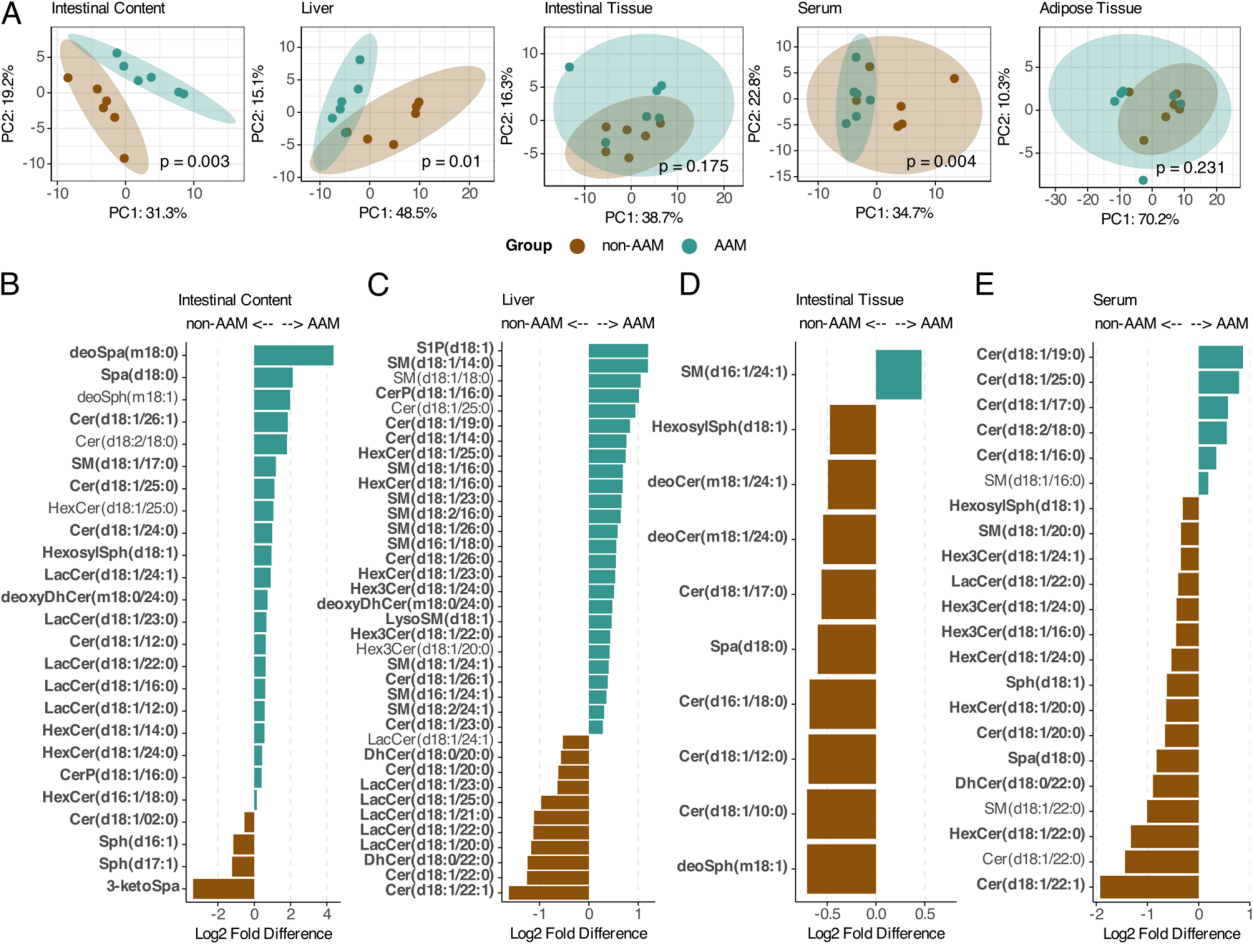
Non-AAM and AAM mice offspring differ significantly in their sphingolipid profiles. **A** PCA plots per organ from the offspring mice based on sphingolipid results. Differences between groups were tested by PERMANOVA (Adonis2 with 999 permutations, non-AAM *n* = 6, AAM *n* = 6). **B**-**E** Metabolites that differed significantly between non-AAM and AAM mice offspring by compartment. T-test, *p* < 0.05, not FDR-adjusted

We continued to investigate whether the AAM and non-AAM groups could be defined by the sphingolipid profile. Indeed, the non-AAM and AAM samples separated significantly from each other in the intestinal content, liver, and serum samples, and there was a slight but nonsignificant trend in the intestinal tissue (Fig. 5A). The results of the control group were similar to those of both groups in most compartments, except for the adipose tissue and intestinal content samples (Supplementary Fig. 6).

A comparison of the sphingolipids between the non-AAM and AAM groups revealed several differentially abundant metabolites (t-test, not FDR-adjusted) that were also previously significantly correlated with the microbiota (Fig. 5B-E).

The murine intestinal content contained several LacCers and ceramides with a d18:1 sphingoid base that were significantly more abundant in the AAM group than in the non-AAM group (Fig. 5B). In the liver, we observed the opposite pattern, with LacCers being significantly lower in the AAM group, while sphingomyelins and ceramides were more abundant (Fig. 5C). In the intestinal tissue, as well as in the serum, the non-AAM group presented higher expression of several sphingolipids (Fig. 5D-E). S1P(d18:1) had significantly greater expression in the AAM group than in the other groups in the liver, but this difference was not detected in any of the other compartments.

### Tryptophan and its metabolites are more abundant in the intestinal tissue of the mice in the non-AAM group

**Fig. 6:**
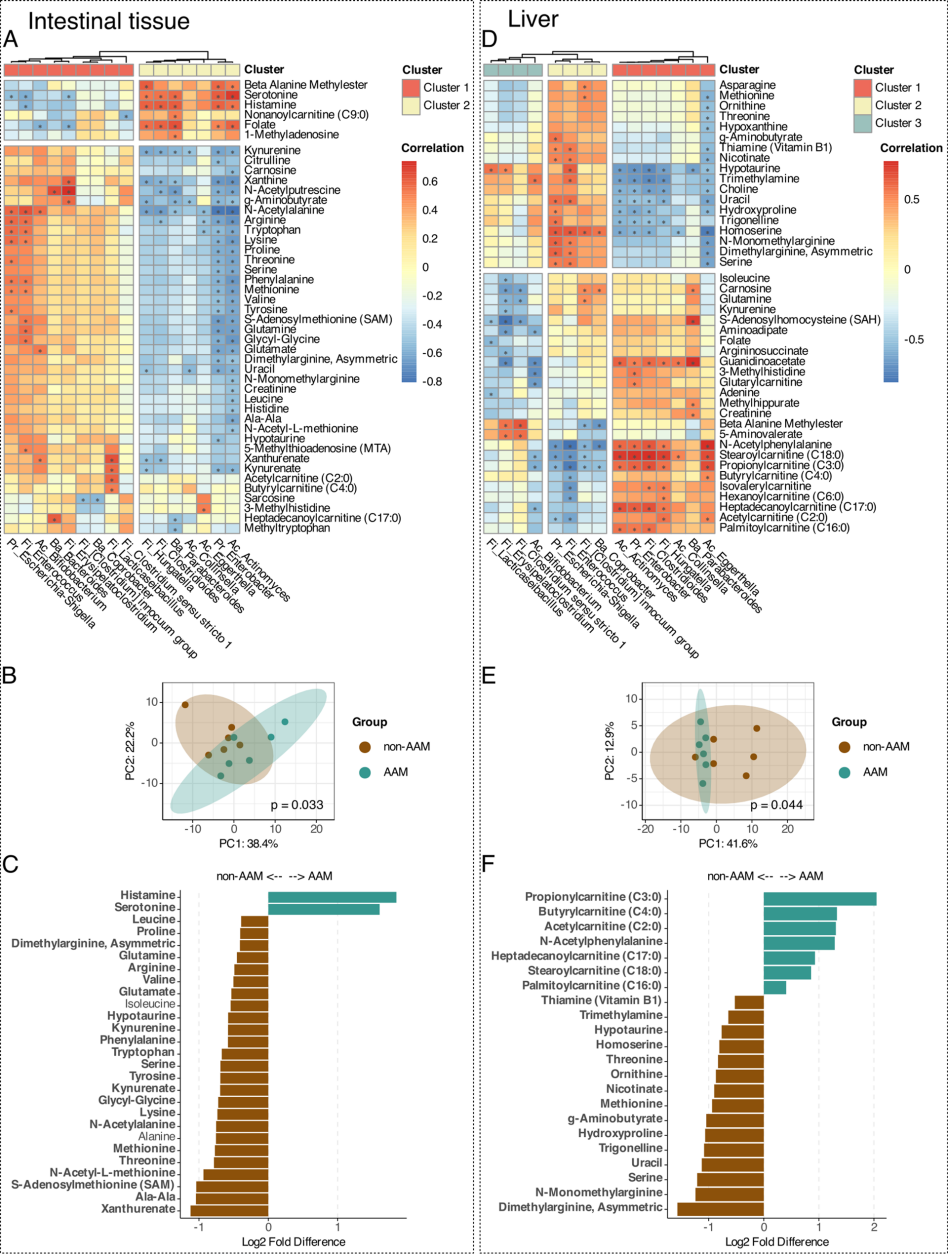
Non-AAM and AAM mice offspring differ significantly in their polar metabolic profiles. Polar metabolite results for the intestinal tissue and liver. **A**, **D** Spearman correlation matrices of bacteria from the mouse offspring at genus level and polar metabolites in the intesintal tissue (**A**) and liver (**D**). Only metabolites and bacteria with significant correlations are shown. Optimal number of clusters are determined using Silhouette method. **B**, **E** PCA on metabolite samples from the intestinal tissue (**B**) and liver (**E**). Differences between groups were tested by PERMANOVA (Adonis2 with 999 permutations, non-AAM *n* = 6, AAM *n* = 6). **C**, **F** Polar metabolites that differed significantly between non-AAM and AAM mice offspring divided by compartment; **C** intestinal tissue, **F** liver. T-test, *p* < 0.05, not FDR-adjusted.

Polar metabolites were measured in the liver and intestinal tissue to identify potential AhR ligands as well as other metabolites with previously reported differences between allergic and non-allergic individuals (Supplementary Fig. 7). We also detected several correlations of these metabolites with the microbiota, which were partially attributable to differences between the non-AAM and AAM groups (Fig. 6).

The microbiota clustered into 2 groups, similar to the clusters observed in the sphingolipid analysis, after correlation with the metabolites in the intestinal tissue. Cluster 1, which included *Lacticaseibacillus*, *Escherichia-Shigella*, and *Enterococcus*, had positive correlations with essential amino acids and tryptophan metabolites. *Actinomyces* and *Enterobacter* in cluster 2 presented strong negative correlations with tryptophan metabolites and amino acids. The majority of cluster 2 bacteria had positive correlations with serotonin and histamine (Fig. 6A). After dividing the samples into the non-AAM and AAM groups, we observed significant clustering by group (Fig. 6B). The metabolites that were significantly more abundant in the non-AAM group were also positively correlated with cluster 1 bacteria and included, but were not limited to tryptophan and several of its metabolites (Fig. 6A, C, t-test, not FDR-adjusted). On the other hand, serotonin and histamine levels were greater in the AAM group than in the non-AAM group and were correlated with cluster 2.

Compared with intestinal tissue, liver tissue presented a different metabolic correlation profile, leading to 3 different bacterial clusters. Cluster 1 in the liver, which included *Eggerthella, Hungatella,* and *Clostridioides,* was the same as cluster 2 in the intestinal tissue. This cluster showed a strong positive correlation with several acylcarnitines and a negative correlation with several essential amino acids (Fig. 6D). After dividing the samples into the non-AAM and AAM groups, we observed significant clustering by group (Fig. 6E). The acylcarnitines that were correlated with the microbiota were also significantly more abundant in the AAM group (Fig. 6D, f, t-test, not FDR-adjusted). We observed similar, albeit nonsignificant, trends for the remaining acylcarnitines (Supplementary Fig. 8). Trimethylamine is produced by gut bacteria from choline [47], and both were significantly correlated with cluster 3 bacteria and were more abundant in the non-AAM group. Apart from acylcarnitines and N-acetylphenylalanine, all other polar metabolites that differed between the two groups were lower in the AAM group than in the non-AAM group in the liver (Fig. 6D, F; Supplementary Fig. 9).

We also performed GC‒MS analysis of the cecal content and liver tissue to investigate short‒chain fatty acids (SCFAs), which revealed higher levels of acetic acid and propanoic acid in the AAM cecal content, whereas butyric acid was not detectable. Only acetic acid was detected in the liver, and no differences were detected between the two groups of mice (Supplementary Fig. 10).

## Discussion

Early gut colonizers, often received from the mother during birth, are crucial in priming the infant’s immune system. In our study, we confirmed the connection between the microbiota-metabolome and immune function, via the use of fecal water preparations from the human infant microbiota to stimulate human PBMCs. We observed group-dependent and allergy-related metabolic characteristics in the fecal water, which led to distinct immune profiles in the PBMCs, mirroring the AAM or non-AAM origin of the inoculum. Next, we used a two-generation HMA model to investigate how the human infant fecal microbiota affects host development in mice, acknowledging the inherent species differences and dietary shifts. The ecological change from humans to mice created a bottleneck effect, where the microbiota was altered, although the extent of this adaptation differed among the three groups, further suggesting that this effect was inoculum dependent. Our study also revealed a significant difference in metabolic profiles between the non-AAM and AAM groups in various compartments, including the liver, intestinal tissue, serum, and the intestinal content. Given that the mice have the same genetic background and diet, differences in metabolic content are most likely driven by the gut microbiota. Taken together, our results point to a clear association between the microbiota composition, the metabolome, and immune profiles, which vary with allergy, and that the HMA model is suitable for studying this connection.

In our HMA model, all groups exhibited a decrease in microbial richness upon introduction into the mice, followed by a slight recovery from the dam to offspring, suggesting adaptation to the new host [13, 48, 49]. Despite this, the groups maintained their unique disparate microbiota profiles from the human inoculum into the mice and across the two generations. This suggests that the microbiota composition and trajectories in the HMA model are donor dependent and that the model is suitable for studying microbiota-host interactions, with relevance to the human inoculates. The observed shift in the abundance of the four major phyla when introduced to the mice aligns with the maturational process in humans, where Actinobacteriota and Proteobacteria decrease, and Firmicutes and Bacteroidota increase during the first year(s) of life [50–52]. This shift is also in agreement with the change of host and diet [10–12, 53]. The observed decline in *Bifidobacteria* in all groups of mice was probably related to the lack of human milk oligosaccharides in the murine diet [54].

Even though we see a shift of the microbiota composition from inoculate to mouse, it was noted that bacteria that are commonly described as immunoregulatory, were vertically transferred to the offspring. For example, *Bifidobacteria* were found in higher relative abundance in both inoculates and murine samples in the non-AAM and control groups compared to the AAM group. In humans, early *Bifidobacteria* colonization is linked to immune imprinting and reduced allergy risk [55, 56]. In addition, a high relative abundance of *Bifidobacteriaceae* or supplementation with *Bifidobacterium* is linked to elevated plasma levels of IL-10 and IL-6 in children and mice, respectively, while a lack of *Bifidobacteriaceae* is associated with increased levels of IL-17A [56–58]. *Bifidobacterium*-derived ILA, which was higher in the non-AAM fecal water, has anti-inflammatory and allergy protective effects on the immature immune system through AhR and TLR-4 [59, 60]. In line with these findings, stimulation of human PBMCs with fecal water from the non-AAM inoculum induced the production of regulatory innate cytokines with high IL-10 and IL-6 levels, while the AAM fecal water additionally induced a type 3 response with a clear IL-17 signature. These results are in accordance with those previously published regarding the immune profile of these mice, with a strong Th17 profile in several immune compartments in the offspring from the AAM group [32].

In an attempt to identify differences in allergy related metabolites, in relation to differences in the microbiota, we started to look into sphingolipids. Sphingolipids are structural components of the cell membranes of both eukaryotes and some prokaryotes and play important roles as signaling molecules that regulate inflammation, asthma, immunity, autophagy, growth, and survival [61–66]. In asthma and allergy, distinct metabolic profiles have been connected to allergic inflammation and airway hyperreactivity to environmental factors [66–70]. The non-AAM and AAM groups separated significantly from each other with respect to the sphingolipid profile in the intestinal content, liver, and serum samples. Several of the observed differences were related to the variance observed in the microbiota, with clear positive and negative associations between the different sphingolipids and gut microbiota species. From a general perspective, clusters of sphingolipids could be identified in the intestinal content as well as in the serum, intestinal tissue and liver, which correlated with some of the microbiota characteristics that initially defined the AAM and non-AAM groups, *e.g., Enterobacter* and *Hungatella,* as well as *Lacticaseibacillus* and *Bifidobacterium* respectively. We also observed that *Bifidobacterium* frequently belonged to the same cluster as *Lacticaseibacillus*. In the liver, for example, several LacCers were more abundant in the group defined by, e.g., *Lacticaseibacillus* and *Bifidobacterium*, and these metabolites were also more abundant in the non-AAM group. In contrast, several sphingomyelins were more abundant in the group defined by, *e.g.*, *Enterobacter* and *Hungatella*, and these metabolites were more abundant in the AAM group.

We also investigated polar metabolites, many of which are associated with allergy and inflammation, as well as immune regulation and homeostasis. This group of metabolites showed several correlations with the microbiota, which were partially related to differences between the non-AAM and AAM groups. Essential amino acids, including threonine, lysine, methionine, homoserine and tryptophan, either originate from the diet or are produced and/or regulated by the gut microbiota [71–73]. These amino acids and their related metabolites, such as S-adenosylmethionine (SAM), kynurenine, and kynurenate, were lower in the AAM mice than in the non-AAM mice. SAM has been shown to protect against tissue damage and fibrosis but also to alleviate allergic inflammation in experimental models [74]. Furthermore, tryptophan and many of its metabolites are essential for intestinal immune homeostasis and gut microbiota maintenance by signaling through AhR [25, 26, 59, 75]. Tryptophan is metabolized either by the microbiota via the indole pathway or by the host itself through the kynurenine or the serotonin pathway [73]. Considering that the only variable in our model is the microbiome, the differences observed in tryptophan metabolites suggest that even endogenous metabolic pathways are influenced by the microbiota.

We also observed that histamine, a major component of tissue mast cell granules and well established as a mediator of allergic symptoms [76], was expressed at higher levels in the intestinal tissue of AAM mice than in the non-AAM mice. Notably, gut microbes can actively produce histamine [77, 78], and adult asthma patients seem to have more histamine-producing microbes than their non-asthmatic counterparts do [79]. In previous reports, acylcarnitines were found to be less abundant in the serum of children with food allergies [70, 80]. We did not study polar metabolites in the serum compartment, but rather observed higher levels of several long acylcarnitines in the liver of the AAM group. The relevance of this for immune priming and allergy development is not clear and needs further investigation, but notably, longer acylcarnitines have been associated with inflammation in other models [81, 82].

Interestingly, the results from the HMA model align well with our initial findings in human samples where PBMCs were exposed to fecal water preparations from the infant inoculates. The lower levels of tryptophan metabolites in the fecal water from the AAM group and the strong immune induction of type 1, 2 and 3 responses are intriguing. Together with the more innate and regulatory immune profile induced by the non-AAM fecal water, these findings reveal a clear link between the microbiome-metabolome and immune profiles.

Our study is not without limitations. Using mice to study the human microbiota creates a bottleneck for certain bacteria that might be of clinical relevance. We used inoculate pools to avoid relying on individual microbiota characteristics, with the result of only three different inoculates used as our starting material. On the other hand, by colonizing the dams, we allowed the microbiota to adjust to the new type of host and were then able to study how mouse-adapted non-AAM and AAM influenced the metabolome in the offspring tissue. Additionally, due to the exploratory character of the study, no adjustments for multiple comparison-false discovery rate were performed. The statistics performed on the metabolomic data was used for hypothesis generation for further testing.

## Conclusion

In conclusion, despite the expected shift in microbial composition between humans and mice, the two-generation HMA mouse model effectively demonstrates the impact of the infant fecal microbiota on host metabolism. Our findings highlight the importance of analyzing both human and murine microbiota to understand the role of microbiota-mediated effects in these models. Even after significant changes post-transfer, the metabolic profiles remained microbiota dependent, suggesting a role for specific bacteria and metabolites in allergy/asthma development. Overall, these findings shows that certain microbiota characteristics modulate hereditary allergy risk, potentially through shifts in the metabolic profile and subsequent immune skewing. Our results emphasize the complex interplay among the microbiota, metabolites, and immune responses in shaping allergy development.

## Supplementary Information

Supplementary Material 1.

Supplementary Material 2.

Supplementary Material 3.

Supplementary Material 4.

Supplementary Material 5.

## Data Availability

The demultiplexed sequence data for 16S rRNA genes have been deposited in the Sequence Read Archive (SRA) of the National Center for Biotechnology Information (NCBI) with BioProject accession number PRJNA1134143.

## Abbreviations

AAM: Allergy-associated microbiota
Ahr: Aryl hydrocarbon receptor
ASV: Amplicon sequence variant
Cer: Ceramide
CLED: Cysteine-, lactose- and electrolyte-deficient
DhCer: Dihydroceramide
ESI: Electrospray ion source
FDR: False discovery rate
GF: Germ-free
HexCer: Hexosylceramide
Hex3Cer: Trihexosylceramide
HMA: Human microbiota – associated
ILA: 3-indolelactic acid
3-ketoSpa: 3-ketosphinganine
LacCer: Lactosylceramide
non-AAM: Non-allergy-associated microbiota
PBS: Phosphate-buffered saline
SAM: S-adenosylmethionine
SCFA: Short-chain fatty acid
SM: Sphingomyelin
S1P: Sphingosine-1-phosphate
SRM: Selected reaction monitoring
