# Supplementary material for: Impact of early-life human microbiota on the murine host metabolome: insights from a two-generation HMA mouse model and implications for allergic disease
Source: BMC Microbiol. 2025 Sep 16;25:575. doi: 10.1186/s12866-025-04321-9 (PMC12439373; doi:10.1186/s12866-025-04321-9)
Supplement: Supplementary file 2 — Supplementary Material 2. [file 12866_2025_4321_MOESM2_ESM.pdf]

Impact of Early-Life Human Microbiota on the Murine Host Metabolome: Insights from a Two-Generation HMA Mouse Model and Implications for Allergic Disease

Ymke A. de Jong[1*], Rana M. Seren[1,2,5], Vida Ramšak Marčeta[1], Antonio Checa[2], Dagbjort H. Petursdottír[1], Isabella Badolati[1], Claudia Moeckel[3], Omneya Ahmed[1], Eva Hell[1], Douglas L. Huseby[4], Diarmaid Hughes[4], Craig E. Wheelock[2,5], Sarahi L. Garcia[6,7], Klas I. Udekwu[8§], Khaleda R Qazi[1§], Eva Sverremark-Ekström[1]

SUPPLEMENTARY FIGURES

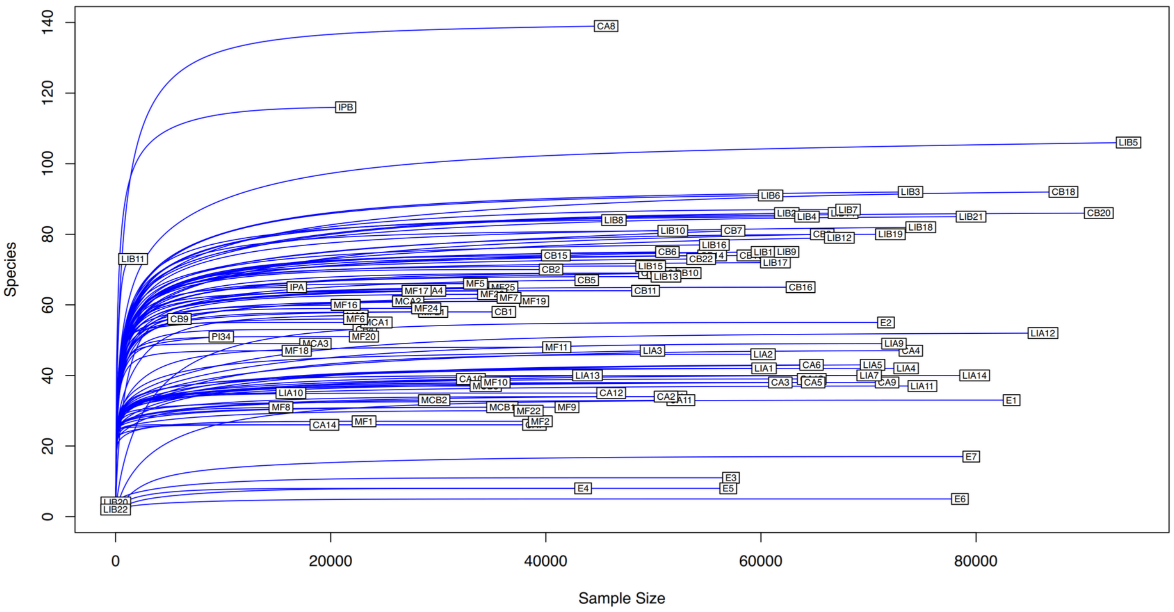

Supplemental Figure 1. Sequence depth per sample. Rarefaction curve showing the difference in sequence depth (Sample size) and number of detected species before rarefying.

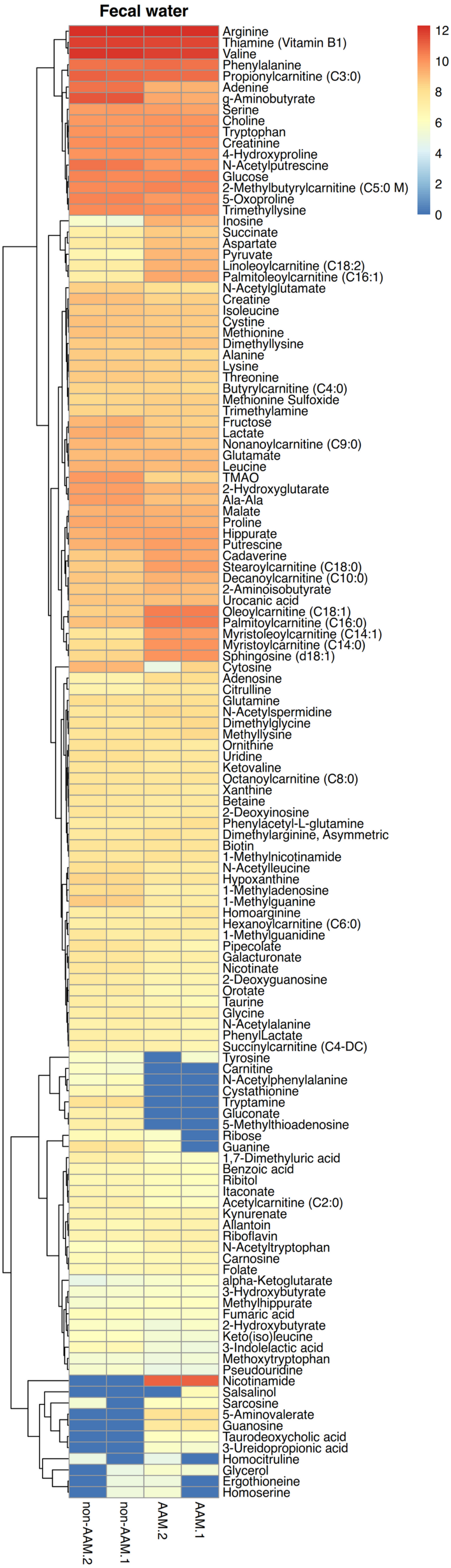

Supplemental Figure 2. Metabolic profile of human infant fecal waters. Metabolic abundances of the human infant inoculi from the non-AAM and AAM group showed as log10 values of the peak area. Each sample was analysed two times.

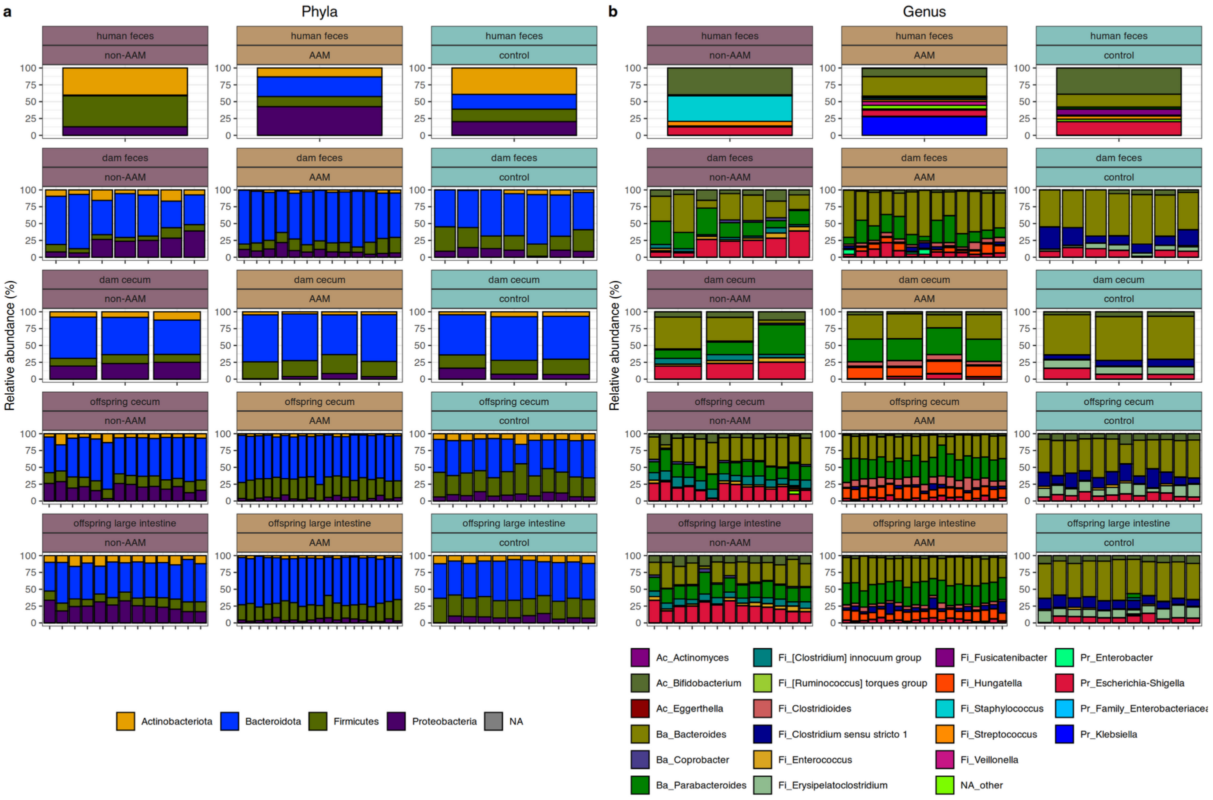

Supplemental Figure 3. Relative abundance of microbial composition of all samples. Composition of each sample showed as relative abundance in stacked bar plots. **a** Bacterial annotation at phyla level with top phyla covering up to 99.9%, remaining 0.1% is annotated as "NA". **b** Bacterial composition per sample annotated on genus level. Mean relative abundance of top genus representing up to 99.5% of total bacteria. Remaining 0.5% are annotated as "NA_other".

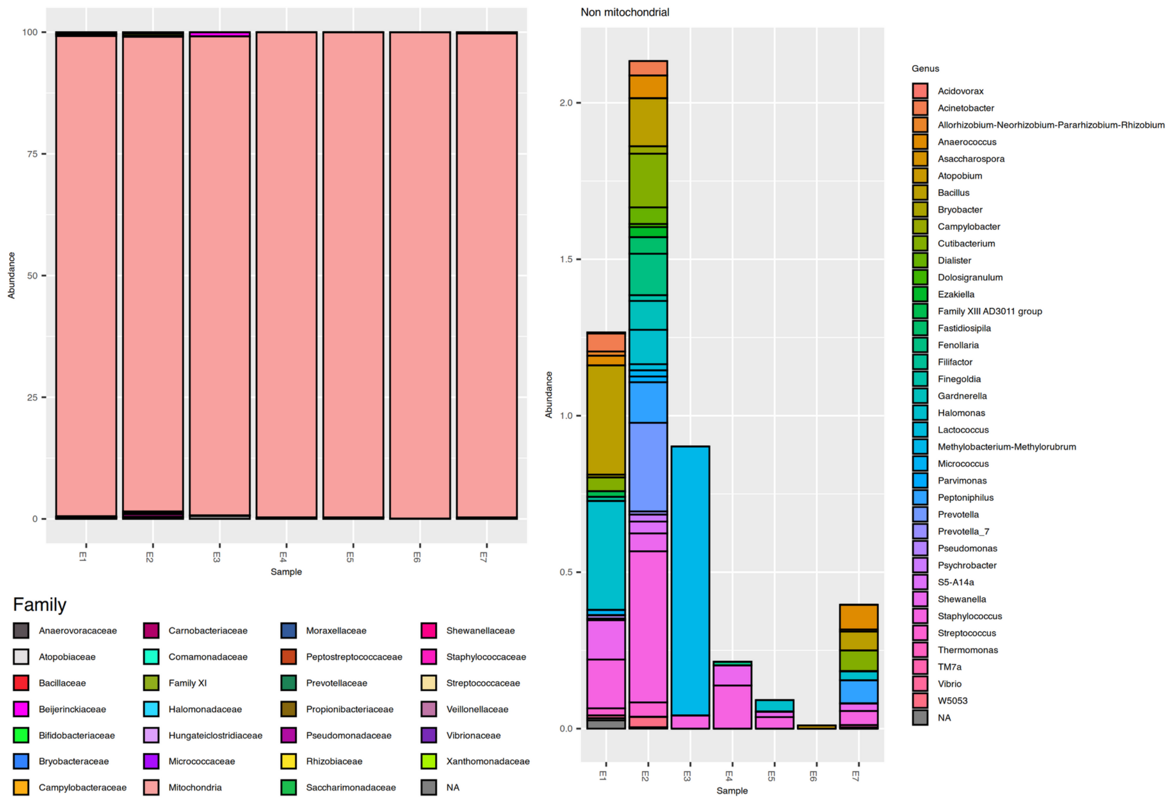

Supplemental Figure 4. Microbiota of seven germ-free mice. **a** Relative abundance of 16s rRNA obtained sequences at family level. **b** Relative abundance of only the non-mitochondrial sequences at genus level.

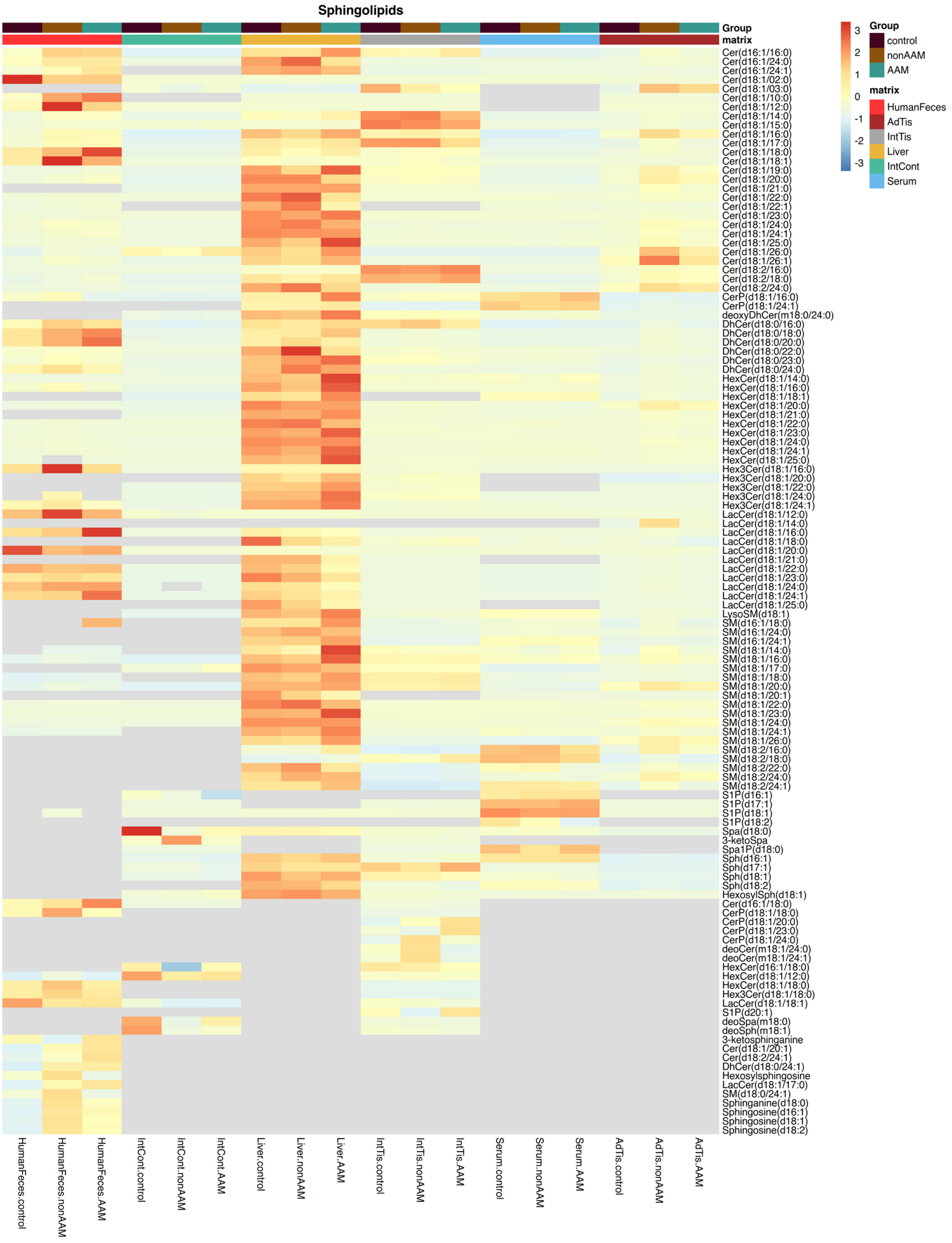

Supplemental Figure 5. Sphingolipid abundances. Sphingolipids measured by LC-MS/MS in human infant feces and each offspring compartment. Scaled by row.

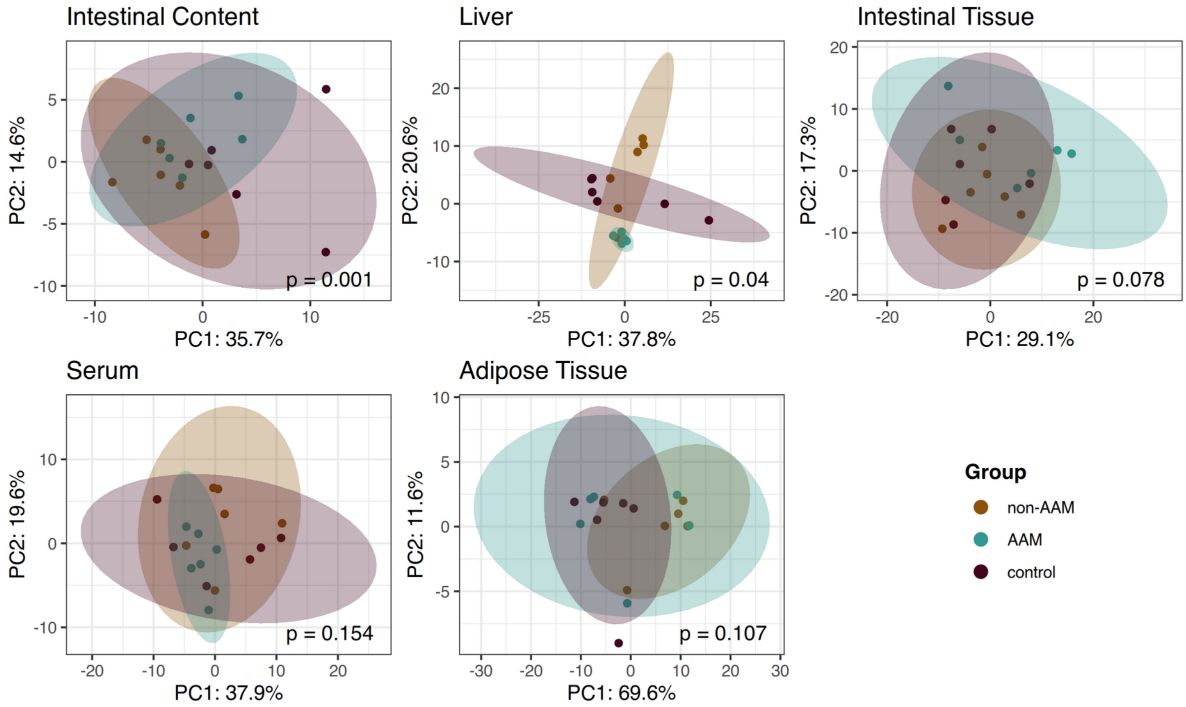

Supplemental figure 6. Sphingolipid profiles of the three groups of mice offspring differ significantly. Differences between groups was tested by PERMANOVA (Adonis2 with 999 permutations, control n = 6, non-AAM n = 6, AAM n = 6).

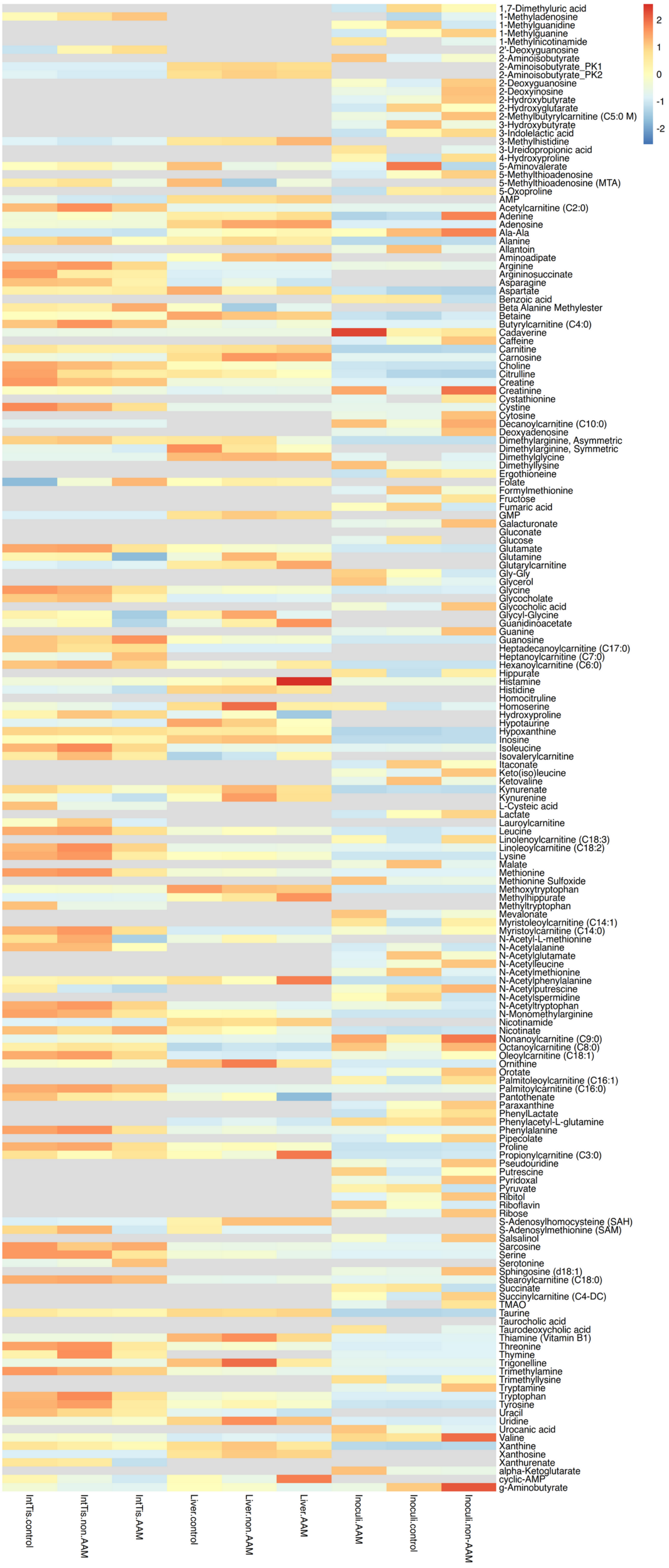

Supplemental Figure 7. Metabolite abundances. Metabolites measured by polar, positive and negative, UPLC-MS/MS in human infant feces, Liver and Intestinal Tissue (IntTis). Scaled by row.

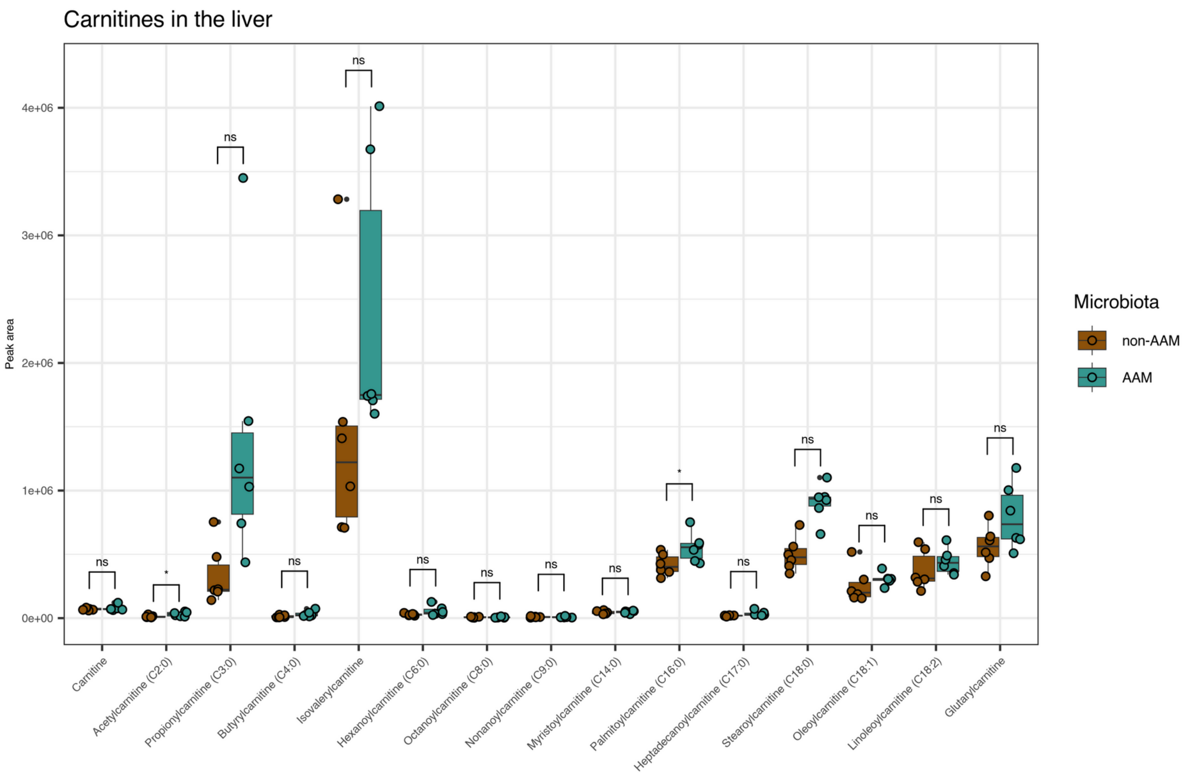

Supplemental Figure 8. Abundance of carnitines in the liver. Peak area of all detected carnitines in the offspring liver. Non-AAM and AAM compared (n = 6, T-test; * $p \leq .05$. Center line corresponds to median, hinges to first and third quartiles and whiskers to min and max (< no further than 1.5*IQR from the hinge)).

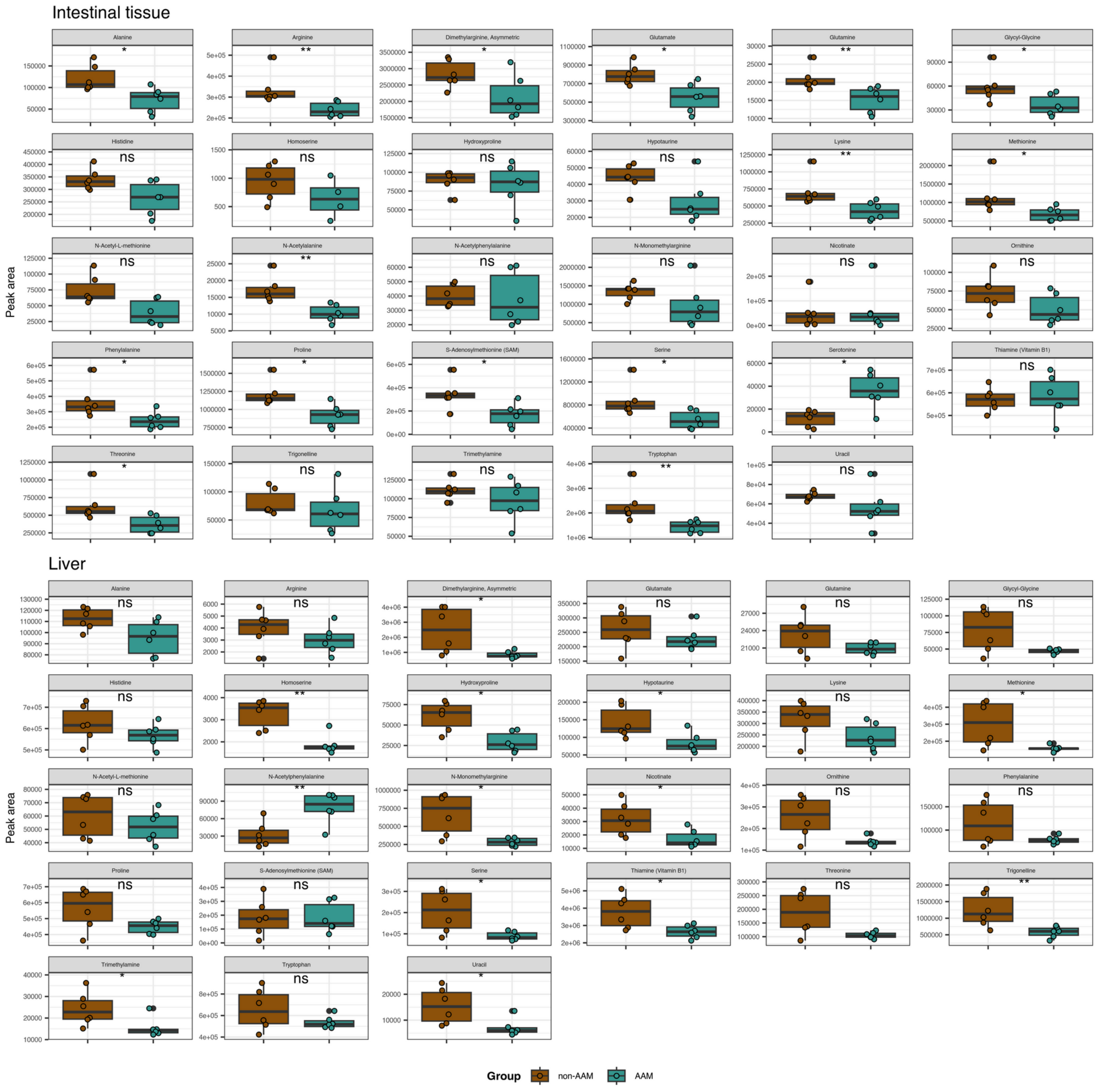

Supplemental Figure 9. Polar metabolites with significant differences between the non-AAM and AAM in either liver or intestinal tissue. Box plots showing the polar metabolites that are significant different between the groups in at least one of the compartments in the offspring. (n = 6, T-test; * p ≤ .05, ** p ≤ .01. Center line corresponds to median, hinges to first and third quartiles and whiskers to min and max (< no further than 1.5*IQR from the hinge)).

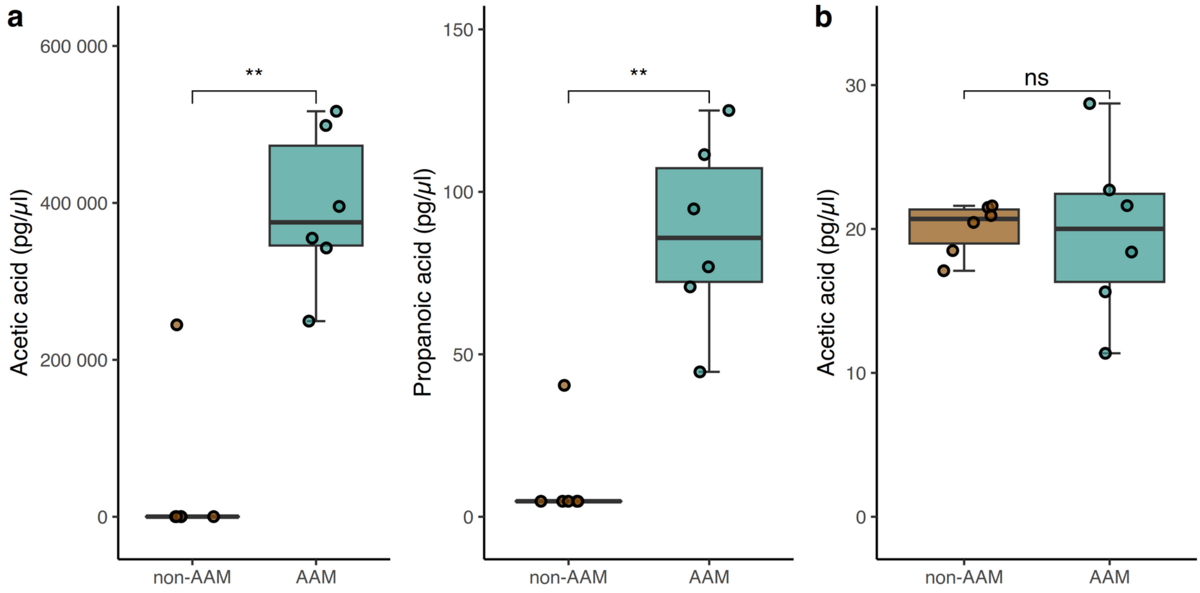

Supplemental Figure 10. SCFA in the cecum content and liver tissue of the offspring mice. Acetic acid, and propanoic acid concentrations measured by GC/MS in **a** cecum content and **b** liver tissue. (n = 6, T-test; ** $p \leq .01$). Center line corresponds to median, hinges to first and third quartiles and whiskers to min and max (< no further than 1.5*IQR from the hinge).
