# Supplementary material for: Impact of early-life human microbiota on the murine host metabolome: insights from a two-generation HMA mouse model and implications for allergic disease
Source: BMC Microbiol. 2025 Sep 16;25:575. doi: 10.1186/s12866-025-04321-9 (PMC12439373; doi:10.1186/s12866-025-04321-9)
Supplement: Supplementary file 3 — Supplementary Material 3. [file 12866_2025_4321_MOESM3_ESM.pdf]

An allergy-associated human microbiota impacts microbial and metabolic profiles in a two-generation HMA mouse model

de Jong YA[1*], Seren RM[1,2,5], Ramšak Marčeta V[1], Checa A[2], Petursdottír DH[1], Badolati I[1], Moeckel C[3], Ahmed O[1], Hell E[1], Huseby DL[4], Hughes D[4], Wheelock CE[2,5], Garcia SL[6,7], Udekwu KI[1,8§], Qazi KR[1§], Sverremark-Ekström E[1]

SUPPLEMENTARY TABLES

Supplementary table 1 GC-MS machine settings

**Column flow**	1.0ml/min
**Injection mode**	Split (50:1)
**Injection temperature**	260°C
**Oven temperature**	40°C for 5 min, 10°C/min to 310°C,310°C for 5 min
**Transfer line temperature**	280°C
**Scan range m/z**	56, 57, 71,85, 99 and 113
**Ion source temperature**	250°C

Supplementary table 2 Analysed m/z-values during SIM mode

**m/z-value**	**Compounds**
56	Acetic acid
57	Propionic acid
71	Isobutanoic acid, Butanoic acid
85	2-methylbutanoic acid, Isovaleric acid, Valeric acid
99	Isopropanoic acid, Hexanoic acid
113	2-Methylhexanoic acid, 4-methylhexanoic acid, Heptanoic acid

Supplementary table 3 DADA2 trimming, quality and merge settings for sequence data preparation. Samples were sequenced on 3 different MiSeq flow cells.

**Function**	**filterAndTrim**			**learnErrors**	**mergePairs**	**assignTaxonomy**
**settings**	**TrimLeft**	**truncLen**	**maxEE**	**Nbases**	**minOverlapp**	**tryRC**
**Dam and Human infant samples ***	21, 50	291, 210	4, 6	3.00E+08	15	TRUE
**Offspring samples**	21, 50	279, 253	1, 2	3.00E+08	15	TRUE
**GF samples**	40, 40	276, 256	2, 2	3.00E+08	15	TRUE

*including CA14, CA7, CA8, CB1
